# Propofol is involved in neurotoxicity by mediating the occurrence of ferroptosis

**DOI:** 10.1097/MD.0000000000043390

**Published:** 2025-07-25

**Authors:** Lei Zuo, Meng Wang, Xin Tian, Lili He, Li Wang, Yunying Li, Xinyu Yao

**Affiliations:** a Department of Breast Surgery, Xingtai People’s Hospital of Hebei Medical University, Xingtai, Hebei Province, China; b Department of Neurology, Affiliated Hospital of Heze Medical College, Heze, Shandong Province, China; c Department of Anesthesiology, Xingtai People’s Hospital of Hebei Medical University, Xingtai, Hebei Province, China.

**Keywords:** ferroptosis, neurotoxicity, propofol

## Abstract

Propofol is the most commonly used intravenous anesthesia in clinical anesthesia and is widely used in various surgeries. Several clinical and preclinical studies have found that propofol can produce neurotoxicity. However, the underlying molecular mechanisms have not been fully elucidated. Ferroptosis is considered to be a new form of cell death. Here, we reveal a new potential mechanism by which ferroptosis is involved in propofol-induced neurotoxicity. We used SH-SY5Y cells as experimental materials to investigate whether propofol produces neurotoxicity by inducing ferroptosis. Our results suggest that propofol significantly causes cytoplasmic iron accumulation through the nuclear receptor coactivator 4-induced ferritin autophagy pathway. Iron overload further induces ferroptosis through the production of lipid reactive oxygen species. Propofol significantly down-regulates the expression of cystine/glutamate antiporter, glutathione peroxidase 4, and glutathione/glutathione disulfide ratio, and up-regulates the expression of acyl-CoA synthetase long-chain family member 4 and NADP^+^/nicotinamide adenine dinucleotide phosphate ratio, which were important makers of ferroptosis. Fer-1, an inhibitor of ferroptosis, could significantly ameliorate the ferroptosis induced by propofol. These data further demonstrate the complexity of the occurrence of propofol-induced neurotoxicity. Inhibiting ferroptosis may be a new strategy for preventing neurotoxicity in the future.

## 1. Introduction

Propofol is an alkyl phenol intravenous injection anesthetic, which was first approved for use in USA in the 1989.^[1,2]^ Propofol has a rapid effect, usually 30 to 40 seconds to produce anesthesia, fast metabolism, but also has the characteristics of rapid and stable recovery, patients can recover consciousness in a short time after withdrawal of the drug, so it is suitable for the induction and maintenance of general anesthesia.^[3–5]^ However, there is growing evidence that propofol has neurotoxic effects on human, raising concerns about brain development by inhibiting the function of hippocampal neurons and possibly leading to neurocognitive decline, although the specific mechanisms remain largely unknown.

As a new type of cell death, ferroptosis is initiated by severe lipid peroxidation, which results from reactive oxygen species (ROS) and iron overload.^[6–8]^ Ferroptosis is characterized by the inactivation or downregulation of glutathione (GSH) peroxidase 4 (GPX4), the inhibition of the light-chain subunit of the cystine/glutamate antiporter (SLC7A11, or system Xc-), and increased levels of free iron and lipid peroxidation.^[9–11]^ As a key regulator of ferroptosis, GPX4 is mainly involved in the repair of lipid damage and the protection of membrane fluid using GSH as a cofactor.^[6,10,12]^ GSH levels are primarily affected by SLC7A11. Inhibition of GPX4 or SLC7A11 can result in ferroptosis.^[10,13,14]^ Due to its close association with neurodegenerative diseases, cancer, and diabetes, ferroptosis has received considerable attention as a potential therapeutic target.^[15–18]^

So far, it has not been known whether the neurotoxicity caused by propofol is related to ferroptosis, so this study aims to explore the relationship between propofol and ferroptosis. We showed here that propofol can induce neurotoxicity in SH-SY5Y cells via ferritinophagy-mediated ferroptosis, indicating that inhibiting ferroptosis is a new strategy for preventing neurotoxicity in the future.

## 2. Materials and methods

### 2.1. Cell culture and treatment

SH-SY5Y human neuroblastoma cells were grown in DMEM supplemented with 10% fetal bovine serum, 100 U/mL penicillin, and 100 mg/mL streptomycin at 37 °C in a humidified 5% CO_2_/95% air incubator. To determine if propofol is neurotoxic and whether it induces ferroptosis, we treated SH-SY5Y cells with different concentrations of propofol or treated the cells with Fer-1 (5 µM) for 24 hours. The experiment was approved by the Ethics Committee of Heze Medical College Affiliated Hospital (Heze City, Shandong Province) (No. 20240615-07) for conduct.

### 2.2. GSSG, GSH, and NADP^+^/NADPH assay

SH-SY5Y cells were tested for GSH and glutathione disulfide (GSSG) concentrations by using the GSH assay kit and GSSG assay kit (purchased from Solabio, China). NADP^+^ and nicotinamide adenine dinucleotide phosphate (NADPH) concentrations were measured by using the assay kit (purchased from Beyotime, Shanghai, China) according to the manufacturer’s instructions.

### 2.3. Assay of cell viability

According to manufacturer’s instructions, SH-SY5Y cells were seeded in 96-well plates to investigate the viability of the cells using the Cell Counting Kit-8 (CCK-8, MCE, China). We added 10 L of the CCK-8 solution per well of the 96-well plates following propofol or non-propofol treatment, and the cells were incubated for 2 to 3 hours at 37 °C. A microplate reader (BioTek, USA) was used to determine the absorbance at 450 nm.

### 2.4. Western blot analysis

We homogenized SH-SY5Y cells in RIPA buffer, centrifuged them at 12,000 *g* for 20 minutes at 4 °C, collected the supernatant containing proteins, and analyzed their protein content using a protein quantification kit (Kang Wei, Beijing, China). After SDS-PAGE, the samples were resolved by 8% to 12%, and then transferred to nitrocellulose membranes (Millipore, Bedford, MA). The target proteins heavy ferritin (FtH) (from Abcam, USA), light ferritin (FtL) (from Abcam, USA), GPX4 (from Abcam, USA), acyl-CoA synthetase long-chain family member 4 (ACSL4) (from Abcam, USA), and nuclear receptor coactivator 4 (NCOA4) (from Abbkine, China) were detected by their primary antibodies. The relative expression quantity of proteins was normalized to that of β-actin (from KangWei, China).

### 2.5. Transmission electron microscopy

We fixed SH-SY5Y cells with 2.5% glutaraldehyde/2% paraformaldehyde (PH 7.2) overnight at 4 °C. Samples were then immersed in 0.1 M sodium cacodylate/HCl buffer (PH 7.2) for 4 hours to remove excess glutaraldehyde. After dehydration through 50%, 70%, 90%, 95%, and 100% alcohol, the samples were embedded in resin and propylene oxide, and finally baked for 48 hours at 65 °C to polymerize. As soon as the samples were polymerized, they were sectioned at 100 nm, lifted onto copper grids of 3 mm in diameter, and stained with 1.5% aqueous uranyl acetate for 30 minutes in lead citrate for a further 10 minutes. The samples were then dried and viewed on the Hitachi H7000 TEM.

### 2.6. Detection of lipid ROS level

Briefly, after the treatment with or without propofol, lipid ROS was examined using the BODIPY 581/591 C11 probe (Thermo Fisher Scientific, Carlsbad). Cells was incubated with BODIPY 581/591 C11 (10 μM) for 30 minutes at room temperature in the dark, washed 3 times with PBS and evaluated using a FACS Calibur flow cytometer (BD Biosciences, CA) to measure ROS production.

### 2.7. Assay of ferrous form (Fe^2+^) content

The iron content was assessed using FerroOrange a (DojinDo, Japan) for the measurement of cytoplasm Fe^2+^. In the assay, ferric carrier proteins will dissociate ferric iron in the reductive environment. After reduction to the Fe^2+^, cytoplasm Fe^2+^ reacts with probes to produce a stable colored complex. The fluorescence intensity was observed using ZEISS LSM510 (LSM510; ZEISS, Germany).

### 2.8. Statistical analysis

The experiments were performed in triplicate and statistical analyses were conducted using GraphPad Software’s Prism 7 (GraphPad Software, USA). Values are presented as mean ± SD. Two-group comparisons were carried out using the Student *t* test (two-tailed). *P*-values < .05 were considered statistically significant.

## 3. Results

### 3.1. Propofol caused SH-SY5Y cells damage in a dose-dependent manner

**Figure 1.**
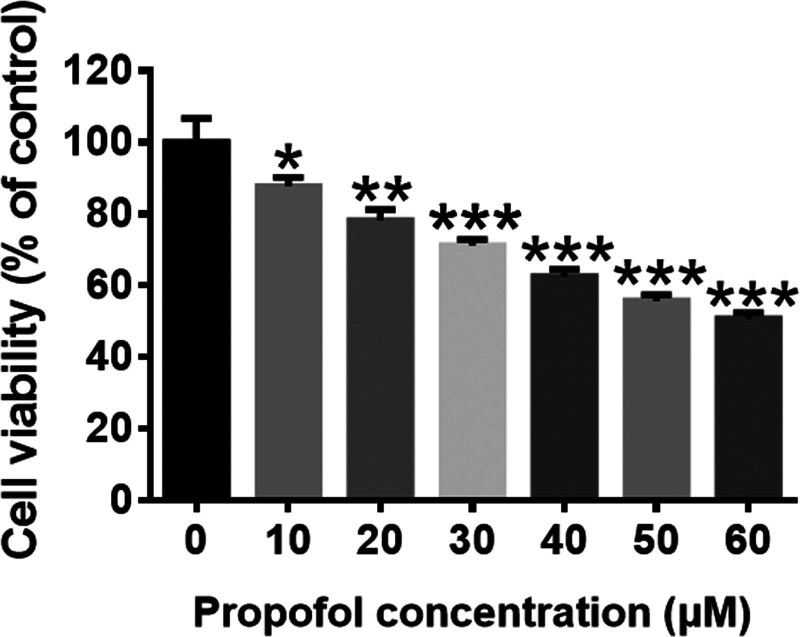
Effects of propofol on SH-SY5Y cell viability. (A) Cell viability was measured by CCK-8 assay (n = 6). SH-SY5Y cells were incubated with or without different concentrations of propofol for 24 hours. Data are presented as the mean ± SD. *P < .05, **P < .01, and ***P < .001 compared with control group.

To determine the concentration of propofol, we used different concentrations of propofol to incubate the SH-SY5Y for 24 hours, then testing the cell viability. The results showed that propofol caused SH-SY5Y cells damage in a dose-dependent manner as shown in Figure 1. A 60 μM propofol reduced cell activity by about 50% compared with that of control group. Therefore, 60 μM was selected as the cell damage concentration for subsequent experiments.

### 3.2. Propofol mediates ferroptosis in SH-SY5Y cells

**Figure 2.**
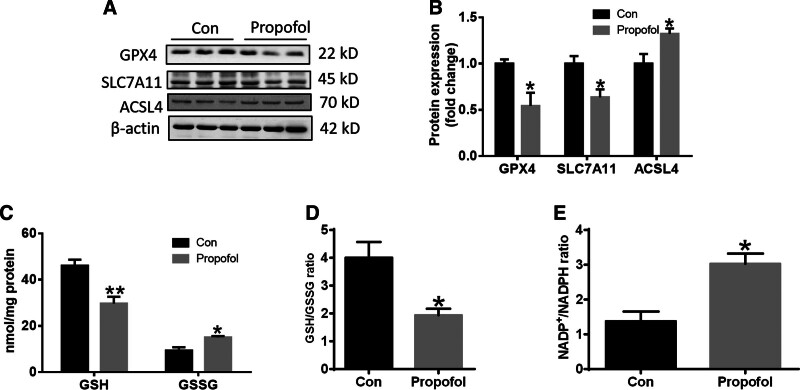
Effects of propofol on SH-SY5Y cell ferroptosis. (A) Western blot analysis of GPX4 and ACSL4 expression in SH-SY5Y cells (n = 3). (B) Quantification of GPX4 and ACSL4 expression from the experiment shown in panel (A). (C–E) The contents or ratio of GSH and GSSG, and the ratio of NADP^+^/NADPH were detected by related kits as described in text of materials and methods (n = 4). Data are presented as the mean ± SD. *P < .05, **P < .01 compared with control group. ACSL4 = acyl-CoA synthetase long-chain family member 4, GPX4 = glutathione peroxidase 4, GSH = glutathione, GSSG = glutathione disulfide, NADPH = nicotinamide adenine dinucleotide phosphate.

Ferroptosis is recognized as a new form of regulated cell death. SLC7A11, GPX4, and ACSL4 are important markers during the process of ferroptosis. In order to verify whether ferroptosis was involved in propofol-induced cell damage, we assessed the expression of the these markers. The results showed that propofol significantly down-regulated the expression of GPX4 and SLC7A11, up-regulated the expression of ACSL4 as shown in Figure 2A and B. Meanwhile, we found that propofol significantly reduced the GSH level and the ratio of GSH/GSSG, and increased the ratio of NADP^+^/NADPH, as shown in Figure 2C–E. These results suggest that propofol induces ferroptosis in SH-SY5Y cells.

### 3.3. Propofol causes iron accumulation in SH-SY5Y cells in the way of ferritinophagy

**Figure 3.**
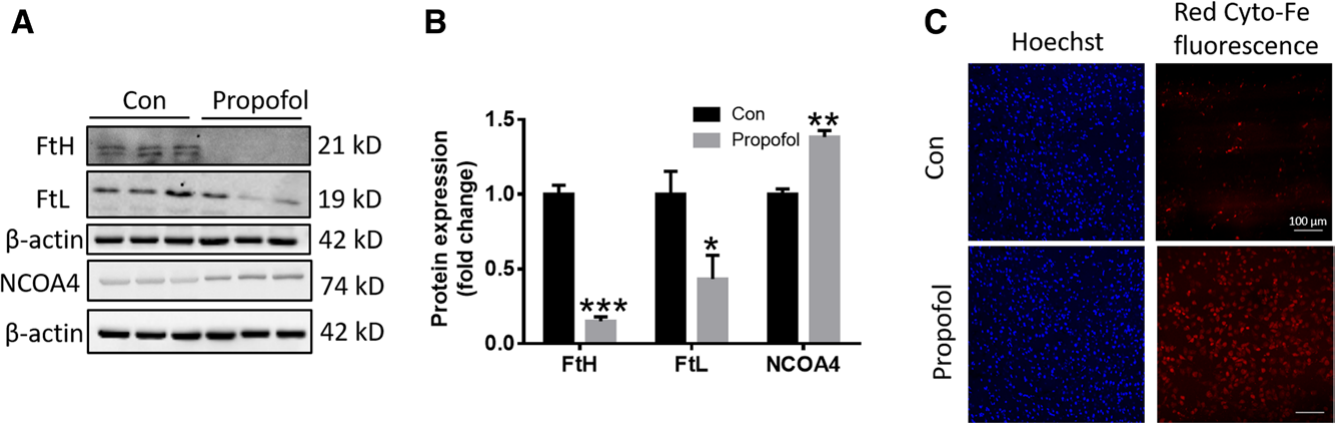
Effect of propofol on intracellular iron content. (A) Western blot analysis of FtL, FtH, and NCOA4 expression in SH-SY5Y cells (n = 3). (B) Quantification of FtL, FtH, and NCOA4 expression from the experiment shown in panel A. (C) Fe^2+^ levels of cytoplasm was detected by fluorescence probe as described in the text of Section 2. Data are presented as the mean ± SD. *P < .05, **P < .01, and ***P < .001 compared with control group. FtH = heavy ferritin, FtL = light ferritin, NCOA4 = nuclear receptor coactivator 4.

Because the essence of ferroptosis is the abnormal metabolism of lipid oxides in cells under the catalysis of iron ions, a large amount of lipid peroxides are produced, which triggers cell death. Therefore, we also examined iron levels in cells after propofol treatment. The results as shown in Figure 3A–C, compared with the control group, the expression of iron storage protein FtL and FtH in cells decreased after propofol treatment, the expression of NCOA4 increased, and the intracellular Fe^2+^ content increased. Ferritinophagy, also known as NCOA4-mediated ferritinophagy, is an autophagic process that releases intracellular free iron.^[19]^ We found that propofol increased the iron levels by initiating ferritinophagy pathway.

### 3.4. Propofol promoted the production of lipid ROS production and damage of mitochondrial

**Figure 4.**
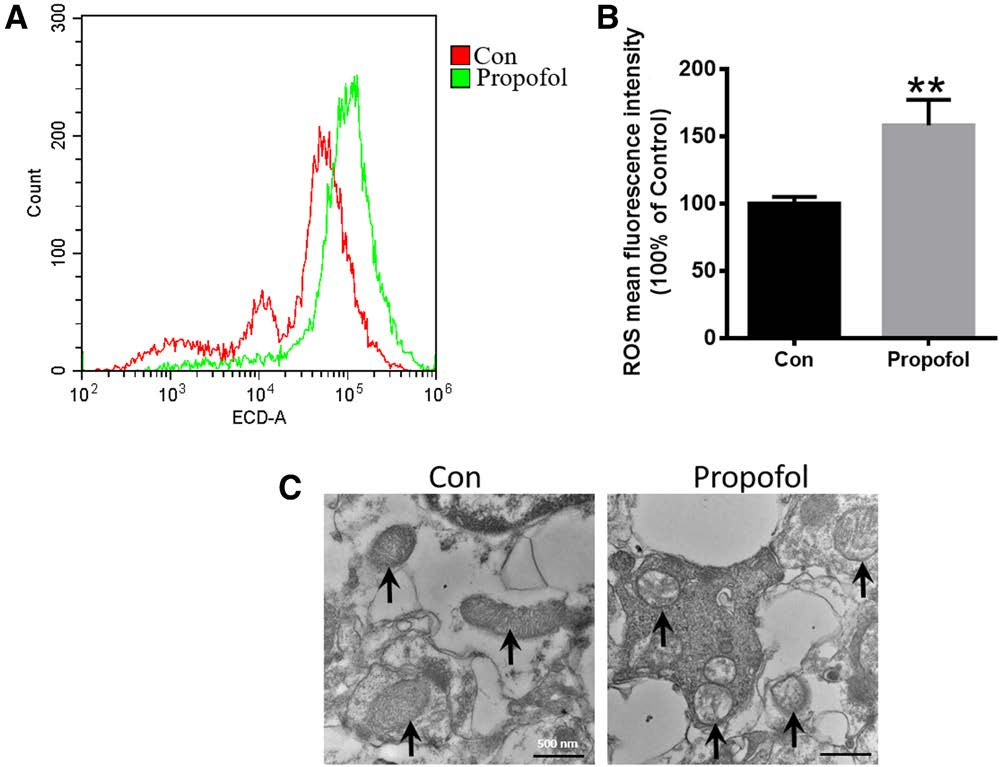
Propofol increased the lipid ROS and damaged the mitochondrial. (A) The lipid ROS was assessed by flow cytometry. (B) Quantification of ROS from the experiment shown in panel (A). (C) The morphological changes of mitochondria were detected by TEM. **P < .01 compared with control group. ROS = reactive oxygen species, TEM = transmission electron microscopy.

Ferroptosis is characterized by significant lipid ROS production and impaired mitochondrial function.^[18,20–22]^ To further verify that ferroptosis contributes to propofol-induced neurotoxicity, we investigated the lipid ROS production and morphological changes in mitochondria in SH-SY5Y cells. As shown in Figure 4A and B, propofol exposure increased lipid ROS levels in the cells compared to the control group. Additionally, mitochondrial morphology was corrupted, as shown in Figure 4C. These findings indeed reveal that propofol caused the ferroptosis in SH-SY5Y cells.

### 3.5. Fer-1 can significantly inhibit the ferroptosis induced by propofol

We performed preliminary experiments with the ferroptosis inhibitor of ferrostatin-1 (Fer-1). As shown in Figure S1A–C, Supplemental Digital Content, https://links.lww.com/MD/P553, compared with propofol group, the cell viability and GPX4 content of propofol + Fer-1 group were significantly increased, while lipid ROS was significantly decreased. These results suggest that Fer-1 (5 µM) can inhibit the neurotoxicity induced by propofol.

## 4. Discussion

At present, the anesthetics used in clinical practice are divided into inhalation anesthetics and injection anesthetics. Inhalation anesthetics include sevoflurane, isoflurane, etc, and injection anesthetics include etomidate, ketamine, propofol, etc. Although the general anesthetic propofol has been used safely in adult surgery for decades, a growing number of preclinical and clinical studies have shown that exposure to general anesthesia in the early stages of neurogenesis can lead to acute and long-term neurotoxicity.^[23]^ The risk of neurological damage is related to the duration, dose, and frequency of exposure to general anesthesia during development.^[24]^

In the present study, we found that high concentrations of propofol (60 μM) did induce damage to SH-SY5Y cells, with a decrease in cell viability of about 50% (Fig. 1). It is suggested that high concentration of propofol can mediate the occurrence of neurotoxicity. Ferroptosis, a form of regulated cell death characterized by the accumulation of lipid hydroperoxides to lethal levels, was linked to degenerative diseases.^[18]^ SLC7A11 is responsible for taking cystine from the extracellular environment and converting it to cysteine in the cytoplasm through a reduction reaction that consumes NADPH.^[25–27]^ Then we use cysteine to make glutathione. The enzyme activity of GPX4 is dependent on glutathione.^[28–30]^ More and more studies have shown that SLC7A11-mediated cystine uptake plays a key role in inhibiting oxidative reaction and maintaining cell survival under oxidative stress conditions.^[16,31,32]^ ACSL4 is induced during ferroptosis.^[33,34]^ Consequently, SLC7A11, GPX4, and ACSL4 are all important markers of ferroptosis.^[35]^ Our study further examined whether ferroptosis affected neurotoxicity induced by propofol by evaluating the expression of these proteins. It showed that ACSL4 expression was increased and GPX4 expression was decreased in SH-SY5Y cells exposed to propofol.

Meanwhile, GSH and NADPH levels, which are responsible for supplying H to GSSG for conversion to GSH, decreased significantly. These results suggest that propofol led to ferroptosis in SH-SY5Y cells (Fig. 2).

One of the important reasons for the occurrence of ferroptosis is due to the accumulation of iron ions, so we also observed the iron level of the cells after propofol treatment. Propofol degrades iron storage protein FtH or FtL through ferritinophagy, releases its stored iron ions, accelerates intracellular iron accumulation (Fig. 3), induces fenon reaction, generates lipid ROS, and damages mitochondria (Fig. 4). Furthermore, the ferroptosis inhibitor Fer-1 significantly reduced lipid ROS accumulation and rescued the decline of GPX4 content (Figure S1, Supplemental Digital Content, https://links.lww.com/MD/P553), suggesting that propofol induces ferroptosis in SH-SY5Y cells. In addition, the limitation of this study is that only propofol mediated ferroptosis was observed at the in vitro level, and there is a lack of in vivo studies. Subsequent studies will be conducted to verify the results of this study.

In summary, our study demonstrates the relationship between high concentrations of propofol and ferroptosis, providing a possible target for the treatment of neurotoxicity due to propofol anesthesia, which has important implications.

## Author contributions

**Conceptualization:** Meng Wang, Xin Tian.

**Data curation:** Li Wang.

**Investigation:** Li Wang.

**Methodology:** Lili He.

**Software:** Xin Tian, Yunying Li.

**Supervision:** Lili He.

**Writing – original draft:** Lei Zuo.

**Writing – review & editing:** Lei Zuo, Xinyu Yao.

## Supplementary Material

## References

[1] Chidambaran V Costandi A D’mello A. Propofol: a review of its role in pediatric anesthesia and sedation. CNS Drugs. 2015;29:543–63. 26290263 10.1007/s40263-015-0259-6 PMC4554966

[2] Briggs LP Clarke RS Watkins J. An adverse reaction to the administration of disoprofol (Diprivan). Anaesthesia. 1982;37:1099–101. 6982636 10.1111/j.1365-2044.1982.tb01753.x

[3] Xuan FL Wang HW Cao LX. Propofol inhibits cerebellar parallel fiber-purkinje cell synaptic transmission via activation of presynaptic GABA(B) receptors in vitro in mice. Front Neurosci. 2018;12:922. 30574067 10.3389/fnins.2018.00922 PMC6291502

[4] Sahinovic MM Struys MMRF Absalom AR. Clinical pharmacokinetics and pharmacodynamics of propofol. Clin Pharmacokinet. 2018;57:1539–58. 30019172 10.1007/s40262-018-0672-3 PMC6267518

[5] Trapani G Altomare C Liso G Sanna E Biggio G. Propofol in anesthesia. Mechanism of action, structure-activity relationships, and drug delivery. Curr Med Chem. 2000;7:249–71. 10637364 10.2174/0929867003375335

[6] Dixon SJ Lemberg KM Lamprecht MR. Ferroptosis: an iron-dependent form of nonapoptotic cell death. Cell. 2012;149:1060–72. 22632970 10.1016/j.cell.2012.03.042 PMC3367386

[7] Mou Y Wang J Wu J. Ferroptosis, a new form of cell death: opportunities and challenges in cancer. J Hematol Oncol. 2019;12:34. 30925886 10.1186/s13045-019-0720-y PMC6441206

[8] Sun S Shen J Jiang J Wang F Min J. Targeting ferroptosis opens new avenues for the development of novel therapeutics. Signal Transduct Target Ther. 2023;8:372. 37735472 10.1038/s41392-023-01606-1 PMC10514338

[9] Song X Long D. Nrf2 and ferroptosis: a new research direction for neurodegenerative diseases. Front Neurosci. 2020;14:267. 32372896 10.3389/fnins.2020.00267 PMC7186402

[10] Latunde-Dada GO. Ferroptosis: role of lipid peroxidation, iron and ferritinophagy. Biochim Biophys Acta Gen Subj. 2017;1861:1893–900. 28552631 10.1016/j.bbagen.2017.05.019

[11] Mao X Liu K Shen S Meng L Chen S. Ferroptosis, a new form of cell death: mechanisms, biology and role in gynecological malignant tumor. Am J Cancer Res. 2023;13:2751–62. 37559994 PMC10408495

[12] Florean C Song S Dicato M Diederich M. Redox biology of regulated cell death in cancer: a focus on necroptosis and ferroptosis. Free Radic Biol Med. 2019;134:177–89. 30639617 10.1016/j.freeradbiomed.2019.01.008

[13] Xu Y Xing Z Abdalla Ibrahim Suliman R Liu Z Tang F. Ferroptosis in liver cancer: a key role of post-translational modifications. Front Immunol. 2024;15:1375589. 38650929 10.3389/fimmu.2024.1375589 PMC11033738

[14] Gong D Chen M Wang Y Shi J Hou Y. Role of ferroptosis on tumor progression and immunotherapy. Cell Death Discovery. 2022;8:427. 36289191 10.1038/s41420-022-01218-8 PMC9605952

[15] Yang F Xiao Y Ding JH. Ferroptosis heterogeneity in triple-negative breast cancer reveals an innovative immunotherapy combination strategy. Cell Metab. 2023;35:84–100.e8. 36257316 10.1016/j.cmet.2022.09.021

[16] Lei G Mao C Yan Y Zhuang L Gan B. Ferroptosis, radiotherapy, and combination therapeutic strategies. Protein Cell. 2021;12:836–57. 33891303 10.1007/s13238-021-00841-y PMC8563889

[17] Dixon SJ Olzmann JA. The cell biology of ferroptosis. Nat Rev Mol Cell Biol. 2024;25:424–42. 38366038 10.1038/s41580-024-00703-5 PMC12187608

[18] Stockwell BR Friedmann Angeli JP Bayir H. Ferroptosis: a regulated cell death nexus linking metabolism, redox biology, and disease. Cell. 2017;171:273–85. 28985560 10.1016/j.cell.2017.09.021 PMC5685180

[19] Tang M Chen Z Wu D Chen L. Ferritinophagy/ferroptosis: iron-related newcomers in human diseases. J Cell Physiol. 2018;233:9179–90. 30076709 10.1002/jcp.26954

[20] Yang WS Stockwell BR. Ferroptosis: death by lipid peroxidation. Trends Cell Biol. 2016;26:165–76. 26653790 10.1016/j.tcb.2015.10.014 PMC4764384

[21] Niu B Liao K Zhou Y. Application of glutathione depletion in cancer therapy: enhanced ROS-based therapy, ferroptosis, and chemotherapy. Biomaterials. 2021;277:121110. 34482088 10.1016/j.biomaterials.2021.121110

[22] Wang B Wang Y Zhang J. ROS-induced lipid peroxidation modulates cell death outcome: mechanisms behind apoptosis, autophagy, and ferroptosis. Arch Toxicol. 2023;97:1439–51. 37127681 10.1007/s00204-023-03476-6

[23] Zhang J Li Y. Propofol-induced developmental neurotoxicity: from mechanisms to therapeutic strategies. ACS Chem Neurosci. 2023;14:1017–32. 36854650 10.1021/acschemneuro.2c00755

[24] Shibuta S Morita T Kosaka J. Effect of preconditioning on propofol-induced neurotoxicity during the developmental period. PLoS One. 2022;17:e0273219. 35984772 10.1371/journal.pone.0273219 PMC9390907

[25] Maschalidi S Mehrotra P Keceli BN. Targeting SLC7A11 improves efferocytosis by dendritic cells and wound healing in diabetes. Nature. 2022;606:776–84. 35614212 10.1038/s41586-022-04754-6

[26] He F Zhang P Liu J. ATF4 suppresses hepatocarcinogenesis by inducing SLC7A11 (xCT) to block stress-related ferroptosis. J Hepatol. 2023;79:362–77. 36996941 10.1016/j.jhep.2023.03.016 PMC11332364

[27] Koppula P Zhuang L Gan B. Cystine transporter SLC7A11/xCT in cancer: ferroptosis, nutrient dependency, and cancer therapy. Protein Cell. 2021;12:599–620. 33000412 10.1007/s13238-020-00789-5 PMC8310547

[28] Bersuker K Hendricks JM Li Z. The CoQ oxidoreductase FSP1 acts parallel to GPX4 to inhibit ferroptosis. Nature. 2019;575:688–92. 31634900 10.1038/s41586-019-1705-2 PMC6883167

[29] Liang D Feng Y Zandkarimi F. Ferroptosis surveillance independent of GPX4 and differentially regulated by sex hormones. Cell. 2023;186:2748–64.e22. 37267948 10.1016/j.cell.2023.05.003 PMC10330611

[30] Xie Y Kang R Klionsky DJ Tang D. GPX4 in cell death, autophagy, and disease. Autophagy. 2023;19:2621–38. 37272058 10.1080/15548627.2023.2218764 PMC10472888

[31] Ye Y Chen A Li L. Repression of the antiporter SLC7A11/glutathione/glutathione peroxidase 4 axis drives ferroptosis of vascular smooth muscle cells to facilitate vascular calcification. Kidney Int. 2022;102:1259–75. 36063875 10.1016/j.kint.2022.07.034

[32] Chen Q Zheng W Guan J. SOCS2-enhanced ubiquitination of SLC7A11 promotes ferroptosis and radiosensitization in hepatocellular carcinoma. Cell Death Differ. 2023;30:137–51. 35995846 10.1038/s41418-022-01051-7 PMC9883449

[33] Li Q Han X Lan X. Inhibition of neuronal ferroptosis protects hemorrhagic brain. JCI Insight. 2017;2:e90777. 28405617 10.1172/jci.insight.90777 PMC5374066

[34] Chen W Han L Yang R. Central role of Sigma-1 receptor in ochratoxin A-induced ferroptosis. Arch Toxicol. 2024;98:3323–36. 38896176 10.1007/s00204-024-03805-3

[35] Gao Z Wang G Chen Y. Total flavonoids of *Astragalus membranaceus* protect against 1-methyl-4-phenyl-1,2,3,6-tetrahydropyridine-induced neurotoxicity in mice by inhibiting ferroptosis through SLC7A11/GPX-4 signaling pathway. Food Sci Human Wellness. 2024;13:414–20.

