# Supplementary material for: Propofol is involved in neurotoxicity by mediating the occurrence of ferroptosis
Source: Medicine (Baltimore). 2025 Jul 25;104(30):e43390. doi: 10.1097/MD.0000000000043390 (PMC12303447; doi:10.1097/MD.0000000000043390)
Supplement: Supplementary file 1 [file medi-104-e43390-s001.doc]

**Supplementary Materials and Methods:**

**Detection of lipid ROS levels**

Briefly, after the treatment with or without propofol, intracellular levels of ROS and lipid ROS were examined using 2',7'-dichlorofluorescein diacetate (H2DCF-DA, Thermo Fisher Scientific) and the BODIPY 581/591 C11 probe (Thermo Fisher Scientific), respectively. Cells were incubated with DCFH-DA (20 μM) or BODIPY 581/591 C11 (10 μM) for 30 min at room temperature in the dark, washed three times with PBS and evaluated using an Enzyme-labeled instrument to measure ROS production.

**GPX4 content assay**

The GPX4 content of cells was carried out using the kit from Mlbio company (NO. ml060706, Shanghai, China) according to the instruction.

**Supplementary Figures and Figure Legends:**


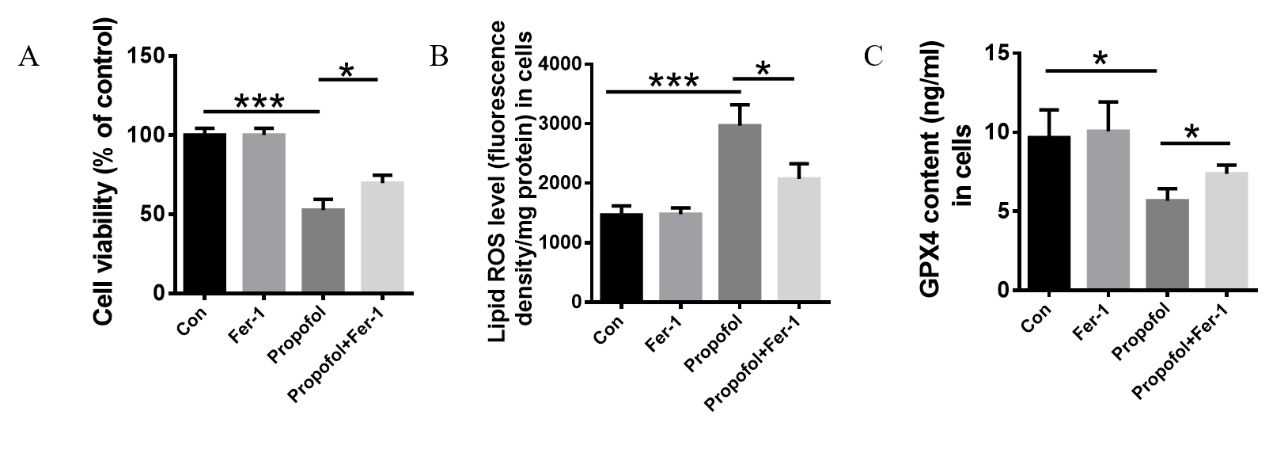


**Figure S1. Fer-1 can significantly inhibit the ferroptosis induced by Propofol.**

(A) Cell viability was measured by CCK-8 assay (n=6). (B) Lipid ROS was tested by fluorescence probe. (C) The expression levels of ferroptosis marker GPX4 were detected by ELISA.
